# Food security and food self-sufficiency around the world: A typology of countries

**DOI:** 10.1371/journal.pone.0213448

**Published:** 2019-03-07

**Authors:** Agnieszka Baer-Nawrocka, Arkadiusz Sadowski

**Affiliations:** Department of Economics and Economic Policy in Agribusiness, Faculty of Economics and Social Sciences, Poznan University of Life Sciences, Poznań, Poland; University of Vermont, UNITED STATES

## Abstract

The particularities of agriculture, as a sector which ensures food supply, result from many factors, including the multilateral interaction between the environment and human activity. The extent of human intervention in the food production process is usually measured with the amount of capital expenditure. Therefore, the food production potential and the resulting food security depend on both natural and economic factors. This paper identifies the current status of food security in different countries around the world, considering both aspects (physical and economic availability) combined together. The variables published by FAO were used together with a variable estimated based on the author’s own methodology to identify 8 groups of countries characterized by economic development level, net trade in agricultural products, and selected variables related to agriculture and food situation. As shown by this study, the degree to which food security is ensured with domestic supply varies strongly across the globe. Domestic production provides a foundation for food security in wealthy countries, usually located in areas with favorable conditions for agriculture (including North America, Australia, New Zealand, Kazakhstan) and in countries which, though characterized by a relatively small area of arable land per capita, demonstrate high production intensity (mainly European countries). International trade largely contributes to food security in Middle East and North African countries as well as in selected South American countries which are net importers of food products. The most problematic food situation continues to affect Sub-Saharan Africa and Central Asia.

## Introduction

Providing food to more than seven billion people living on the planet is among the key challenges for today’s world and one of the Millennium Development Goals (MDGs) [1–4]. Since the dawn of humanity, people have been making continuous efforts to remain food secure, and the capacity to feed the population has long drawn intense interest from the scientists. Many studies have evaluated that problem, e.g. [5–11]. However, food security entered the socio-economic dictionary only in the 1970s [12]. Since then, it has been systematically revised, as reflected in the number of existing definitions, e.g. [13–16]. The initial approaches to food security focused on food stocks which allowed to survive famine. As the level of overall human development was rising, another reason for food insecurity was found to be the insufficient purchasing power of poorer population segments. Finally, health qualities and nutrient content of food became a matter of concern for food security. These three aspects, i.e. physical and economic availability and food safety and quality, as well as the stability of all these dimensions over time, are addressed in the most commonly used definition, as provided for in The State of Food Insecurity in the World 2001 report [17]. In the literature, food security is usually considered at three levels: the farm level, the national level and the international level. All of these dimensions are interconnected, and therefore form a set of targets which are often difficult to extract. As emphasized by Srinivasan [18] and Dawe [19], in addition to price and disposable income, food security at household level is also largely affected by: the rural population’s level of and access to education (especially as regards women and poor); child healthcare; food education; consultancy on how to manage farms and set up kitchen gardens. At national level, food security is generally assessed based on actual average energy intake per capita in relation to the needs which are determined in accordance with minimum recommended nutrition standards. Usually, food balance sheets are used for that purpose [20]. The selection of a national food security strategy depends on production resources and on the systemic and institutional condition of the political, economic and social life of a country. In that context, there are three main types of agri-food policy solutions aimed at food security. Specifically, this means efforts taken to ensure [21]: food self-reliance, food self-sufficiency and food sovereignty. While the assumption of the first strategy is to produce and export goods in which the country has a comparative advantage (which provides many opportunities, including the generation of financial resources and imports of other agricultural products), the two other are based on enhancing the domestic production of basic agricultural products, though the country has no comparative advantage in it. As emphasized by Pieterse et al. [21] what matters in food sovereignty is not only the right to food but also the right to produce food. That concept focuses on the role of family farming, organic production methods and a fair distribution of productive inputs. The strategy based on food self-sufficiency (which limits the role of imports to food products) became increasingly important during the last economic crisis. At that time, many countries found it to be one of key priorities for their agri-food policies.

International (global) food security is related to social efforts taken to reduce excessive regional disparities in combating hunger. This issue may be considered in a broader and narrower sense. In that sense, food security is mainly limited to food stocks, primarily including cereals which are the basic product used to meet the nutritional requirements around the world. In a broader sense, it involves many components of the food system which includes food production and distribution, food aid, food stocks, production and consumption information systems, and programs implemented to improve the food situation of the world’s population.

Problems related to the nutritional status of the population in different parts of the world are extensively reflected in literature. Many papers emphasize that despite the growing supply of food, the problem of ensuring food security on a global basis has not yet been solved [22–28]. It is also noted that because of today’s production scale and intensity, the limits of the Earth’s capacity to provide a safe place for humans have been reached [29,30]. In that context, another important aspect is the environmental impact of agricultural production, including climate change [31–35]. Much attention is also paid to various scenarios for the future development of food production and consumption around the world. They predict a further increase in food demand and an increase in the share of animal products in the diet, especially in developing countries, e.g. [25,36,37]. Food security issues are quite often considered on a countrywide basis or within country groups [38–41] or at a world-wide level [42–45]. Nevertheless, because of its importance, that topic still needs to be analyzed and observed on a continuous basis, and therefore leaves much to be discovered.

In practice, the main responsibility for food security and self-sufficiency is the agricultural sector which is supported to a small extent by hunting and fisheries. The particularities of agriculture result from many factors, including the specific, multi-faceted interaction between the environment and human activity. This means the capacity to produce an adequate volume of agricultural raw materials is affected, on the one hand, by natural conditions (primarily including soil and climate) and, on the other, by anthropogenic factors, such as progress in organization and in technical, chemical and biological sciences. As the agriculture develops, human impact becomes increasingly important, although the natural environment continues to play a major role. In that context, note that the scale of human intervention in food manufacturing processes is roughly measured with the amount of capital expenditure, i.e. it depends on the economic development level of a country. Ultimately, the food production capacity and the resulting food security are determined by natural and economic factors. In view of the above, the purpose of this paper is to determine the differences between countries around the world while taking into consideration both food security and food self-sufficiency, and then to characterize the groups of countries by economic development level, net trade in agricultural products, and selected variables related to agriculture and food situation. This will allow to identify the current status of food security in different countries around the world (based on latest available data), considering both aspects (physical and economic availability) combined together. This paper adds value by providing a global approach to food security, addressing both production and consumption aspects. In that respect, the approach proposed in this paper differs from other analyses. For instance, Porka et al. [42] developed three independent classifications of the world’s countries based on three important food security indexes, i.e. food availability, food self-sufficiency and food trade. Moreover we emphasized the natural and economic determinants of the geographic heterogeneity of developments under consideration. This paper mainly addresses the agricultural production capacity of different countries covered by the study, expressed in kcal produced per hectare of UAA. The production capacity was compared against the degree to which food needs are met. Therefore, an important advantage of this study is a synthetic approach to production and consumption issues, enabled by the use of a synthetic indicator which takes kcal produced and consumed into account, and provides a basis for the typological classification of countries around the world. This allowed to diagnose the areas which differ from one another in both aspects: the food production capacity and food consumption.

We are aware that because of the approach used and of its global nature, this study fails to take account of a number of major aspects of food security, such as the supply of adequate quantities of proteins and other nutrients [46], or the country-level variation in the aspects considered. Extending our analysis to examine such issues is a good topic for further research. However, the main goal of this approach was to emphasize the importance of basic geographic, natural and economic factors in ensuring food security and food self-sufficiency at a global level. In this context, the authors’ approach is consistent with the requirement set out by Coates [47] who assumed that a more holistic and inter-sectoral approach to the food problem needed to be adopted. The importance of analyses based on a comprehensive system perspective is also emphasized by Puma et al. [44]. As noted by Peréz-Escamilla et al. [48], the identification of food security indexes based on various methodological assumptions is important for improving the effectiveness, development and sustainability of an adequate food security governance system at a local through to a global level.

## Materials and methods

### Data sources

Nutrition and food security are the overarching objectives of the Food and Agriculture Organization of the United Nations (FAO), and therefore a large part of available data and materials concerning this issue is published by FAO. We use the following variables from the FAOSTAT database: population (million people), agricultural area (hectares), net trade in agricultural products (USD billion). FAO also delivers Food Security Indicators report [49] a set of indicators comparable between regions around the world which are designed to capture various aspects of food insecurity. Among them, the following indicators were used for analytical purposes in this paper:

- Dietary Energy Supply (DES) (kcal/capita/day),
- Average Dietary Energy Supply Adequacy (ADESA), which expresses the Dietary Energy Supply (DES) as a percentage of the Average Dietary Energy Requirement (ADER) (%)
- number of people undernourished (million people).

All the variables listed above, just like all other used in the analysis, were defined as the mean level recorded in 2000–2013. This approach was adopted to avoid one-year fluctuations affecting the developments under consideration.

### Determining the production volume of agricultural energy

From the perspective of the purposes of our analysis, the Average Production of Agricultural Energy (APAE), expressed in kcal, was an important variable. It was estimated based on the author’s own methodology, with the use of the following algorithm:

$$APAE=\frac{\sum_{i=1}^{n}{ES}_{i}*W_{i}}{365}$$ (1)

where:

APAE: Average Production of Agricultural Energy (kcal /capita/day)

Es_i_: average energy consumption of agricultural product i (kcal /capita/year)

W_i_: food self-sufficiency ratio for product i, calculated as:

$$W_{i}=\frac{P_{i}}{Z_{i}}$$ (2)

where:

P_i_: production volume of product i (tons /country/year)

Z_i_: domestic supply quantity of product i (tons/country/year)

APAE is estimated using multiple factors, including selected items of the food balance sheet established for specific products as per the FAO methodology. The objective of this approach was to convert the agricultural (both crop and livestock) production volume into a single, universal energy unit. The following products were included: meat, milk, offals, eggs, cereals, starchy roots, pulses, oilcrops, vegetable oils, sugar crops and sugar, fruits, vegetables, stimulants, species, treenuts, alcoholic beverages.

Domestic supply quantity (Z_i_) in the algorithm (2) was calculated as: production + import + stock variation–export. In this sense, the method employed in this study is consistent with that used by FAO to calculate the self-sufficiency ratio (SSR), which is defined as the percentage of food consumed that is produced (SSR = Production x 100 / (Production + Imports–Exports)). More precise measurements of the SSR also include changes in domestic stock levels [45]. The coefficients were calculated by us because the unpublished mean values for 2000–2013 needed to be used in the conducted analysis.

The study covered all countries around the world with a population above 1 million for which essential statistical data was readily available. Countries with a population below 1,000,000 are of minor global importance. Because of their particularities, agriculture plays a marginal role for most of them. Some of these countries are cities (e.g. Monaco, Vatican) while other focus on one particular sector (e.g. tourism in Belize). Therefore, the assumption was made that in these countries, the relationships between food production and consumption are of a different nature than in more populated countries. See the “Supporting information” section (S1 Table) for the list of countries covered by this analysis (grouped by classes).

### Typology of the countries

As the first step, a typology of countries was developed based on two basic criteria. The first one took into consideration the ADESA level to identify two classes:

- ADESA >100%: class 1
- ADESA < 100%: class 2

This is how we wanted to identify the countries where the actual daily intake is higher (class 1) or lower (class 2) than the average dietary energy requirement.

The second classification criterion was the difference between the Dietary Energy Supply (DES) and the Average Production of Agricultural Energy (APAE). Similarly, two classes were defined:

- APAE (kcal /capita/day) > DES (kcal/capita/day): class 1
- APAE (kcal /capita/day) < DES (kcal/capita/day): class 2

This allowed to identify the countries where the average production per capita was higher (class 1) or lower (class 2) than the consumption level.

Ultimately, considering both criteria, four basic typology classes were established:

1.1: ADESA > 100% and APAE (kcal/capita/day) > DES (kcal/capita/day). Countries where the consumption level is at least equal to the average dietary energy requirement and where domestic production fully covers the energy intake. In this group, countries do not face any serious food problems and are food self-sufficient.
1.2: ADESA > 100% and APAE (kcal/capita/day) < DES (kcal/capita/day). Countries where the consumption level is above the average dietary energy requirement but domestic production is not enough to address the food requirements of the population. While these countries remain food secure, they are not fully self-sufficient in food.
2.1: ADESA < 100% and APAE (kcal/capita/day) > DES (kcal/capita/day). Countries where the average intake is lower than average food requirements but the agricultural energy production volume per capita exceeds the energy intake. This means that despite severe food problems, they decide to export agricultural products for various reasons.
2.2: ADESA < 100% and APAE (kcal/capita/day) < DES (kcal/capita/day). In these countries, too, the intake does not match the average food requirements. On top of that, the agricultural energy production is lower than energy consumption. This means these countries suffer from food insecurity combined with the lack of food self-sufficiency.

A preliminary analysis showed that classes 1.1 and 1.2 were the largest ones and included countries with different economic characteristics. Therefore, later in the analysis, median GDP per capita was used as a criterion to identify two sub-classes in each class. GDP per capita figures were retrieved from the World Bank database. The final result is as follows:

1.1.1: ADESA > 100%, APAE (kcal/capita/day) > DES (kcal/capita/day), GDP per capita > median
1.1.2: ADESA > 100%, APAE (kcal/capita/day) > DES (kcal/capita/day), GDP per capita < median
1.2.1: ADESA > 100%, APAE (kcal/capita/day) < DES (kcal/capita/day), GDP per capita > median
1.2.2: ADESA > 100%, APAE (kcal/capita/day) < DES (kcal/capita/day), GDP per capita < median

Based on the above, prosperous and less prosperous countries were extracted from the two largest groups, which ultimately enabled the identification of both natural/geographic and economic factors affecting food security and food self-sufficiency.

The subsequent part of the analysis consisted in characterizing the classes and subclasses by selected features relating to their economy, agriculture and food situation. In the case of relative indicators, the following algorithm was used:

$$Cb=\frac{\sum_{t=1}^{n}\sum_{i=1}^{n}CZ1}{\sum_{t=1}^{n}\sum_{i=1}^{n}CZ2}$$ (3)

where:

*Cb*: feature under analysis

*i*: countries grouped in class i

t: successive years from 2000 to 2013

*CZ1*: first factor defining the feature under consideration (e.g. agricultural energy production volume, GDP/capita),

*CZ2*: second factor defining the feature under consideration (e.g. agricultural land area, population).

For the diagram of the research process see the “Supporting information” (S1 File).

## Results

### Importance of countries of different classes and subclasses

As shown by the analyses, a vast majority of the human population live in countries where the agricultural output, expressed in kcal, is below the consumption level: nearly 75% of the global population live in countries grouped in class 1.2 (Table 1, S1 Table). Therefore, it may be concluded that the agricultural production potential is unevenly distributed across the globe, and that some regions are “global granaries” because of their natural conditions and capital expenditure. Generally, this is driven by two factors. The economic factor is important for food production, all the more so since 32 states grouped in class 1.1 are inhabited by only 22.5% of the human population. However, these countries generate as much as 42% of the global GDP. Note however that this results mainly from the considerable wealth of states grouped in subclass 1.1.1. A large group of countries constituting subclass 1.1.2 (generally characterized by a smaller share in the global GDP than in the population) proves that the geographic and natural factors continue to play an important role for food production. Though relatively less wealthy (if not poor), these countries record a surplus on their agricultural markets. This, however, largely results from favorable natural conditions. On the other hand, the results of this study show the strategic importance of food security and self-sufficiency. For each class and subclass, note that the gap between the share in the population and the share in agricultural energy output is definitely narrower than between the share in the population and the share in GDP. Obviously (in accordance with the delimitation assumptions), class 1.1 countries have a larger share in energy output than in the population whereas it is the opposite for class 1.2. However, it is still noticeable that all countries make efforts to ensure an adequate production output within their territory, depending on their economic and natural potential.

**Table 1:** Importance of countries grouped in specific classes and subclasses on a global scale (2000–2013 average figures).

Class	Subclass	Number of countries	Population (World = 100)	Gross Domestic Product (World = 100)	Production of agricultural energy (APAE) (World = 100)
1.1. ADESA>100% APAE> DES	Total	32	22,5	42,0	31,8
	1.1.1. GDP/capita > median (15662$)	16	10,7	33,8	16,9
	1.1.2. GDP / capita < median (15662$)	16	11,7	8,2	14,9
1.2. ADESA>100% APAE< DES	Total	87	74,8	57,6	66,5
	1.2.1 GDP / capita > median (8640$)	44	19,2	35,4	16,4
	1.2.2 GDP / capita < median (8640$)	43	55,7	22,2	50,2
2.1. ADESA<100% APAE> DES	Total	2	0,3	0,1	0,3
2.2. ADESA<100% APAE > DES	Total	14	2,4	0,4	1,4

Source: own calculations based on [49,50]

Class 2.1 and 2.2 countries are a separate problem. While their share in the global population is up to 3%, the fact alone that they face serious food problems (reporting an average dietary energy supply which does not fully address the demand) should be a challenge not only for the local societies but for the whole human kind. Note also that while all classes and subclasses include countries affected by undernourishment (which will be subject to further analysis), the problem is extremely severe only in class 2.1 and 2.2 countries.

While the location of countries grouped in different classes and subclasses does not provide a clear basis for establishing their natural and geographic characteristics, some patterns are noticeable (S1 Table). Class 1.1 countries are located across all continents but are mostly concentrated in North America and Europe. Usually, they demonstrate either favorable agricultural conditions or high (class 1.1.1) or medium (class 1.1.2) levels of wealth (which translates into capital investments into agricultural production), or both. Class 1.2 countries are also located in different parts of the world, but are essentially characterized by adverse natural conditions affecting their agricultural sectors. Many of them are located in arctic or desert environments. This justifies the agricultural production deficit which is recorded in each of these countries irrespective of their wealth. Note however that 1.2.1 class countries include European countries and oil exporters from the Middle East. In turn, class 1.2.2 is mainly composed of quite poor Asian and African Sub-Saharan countries. This is also true for classes 2.1 and 2.2.

### Characteristics of country classes identified

As a next step, this study included preparing the characteristics of different country classes and subclasses in terms of food production and consumption and of underlying natural, geographic and economic factors.

The largest daily energy supply per capita is recorded in countries with the highest GDP per capita, i.e. members of classes 1.1.1 and 1.2.1 (Table 2). These groups are also characterized by the lowest share of the undernourished population, even though the members of the first subclass have a surplus agricultural production capacity while the second subclass consists of net importers. Classes and subclasses with lower average income levels demonstrate lower consumption levels and larger shares of the undernourished population, with the worst situation being in classes 2.1 and 2.2. We can conclude that in a globalized economy, an adequate amount of capital is enough to meet the food requirements even if a country does not have favorable conditions for agricultural production.

**Table 2:** Production, economic and natural aspects of food security in classes and subclasses identified (2000–2013 average figures).

Class	Subclass	GDP per capita (USD)	Net trade in agricultural products (USD billion)	Arable land per capita (ha)	Production of agricultural energy (thousand kcal/ha)	Production of agricultural energy (kcal/person/day)	Daily energy supply (kcal/person)	Difference between production of agricultural energy and daily energy supply (kcal/day)	Share of the undernourished population (total population = 100)
1.1. ADESA>100% APAE> DES	Total	23113	133,2	1,3	2963	3826	3106	720	6,6
	1.1.1. GDP/capita > median (15662$)	39241	77,8	2,0	2121	4267	3507	761	0,5
	1.1.2. GDP / capita < median (15662$)	8557	55,5	0,6	5397	3424	2745	679	12,1
1.2. ADESA>100% APAE< DES	Total	9538	-141,6	0,5	4646	2397	2769	-372	13,6
	1.2.1 GDP / capita > median (8640$)	22708	-120,9	0,8	2858	2297	3153	-856	3,3
	122 GDP / capita < median (8640$)	4957	-20,6	0,4	5836	2431	2636	-205	17,2
2.1. ADESA<100% APAE> DES	Total	3098	0,4	2,0	1148	2288	2136	152	37,0
2.2. ADESA<100% APAE> DES	Total	1993	-1,0	2,4	662	1606	2081	-475	40,1

Source: own calculations based on [50,51].

As regards agricultural production, other patterns were observed. First of all, note that the surplus or deficit is characteristic for both energy indices (defined as the difference between energy production and consumption per capita) and economic indices (expressed as the net trade in agri-food products). This suggests that an approach based on natural energy indices would be reasonable. Countries where output exceeds consumption have two essential characteristics. Firstly, they are quite wealthy; this is true not only for class 1.1.1 composed of the wealthiest countries of the world. In class 1.1, the average income per capita is much higher than in other classes; in subclass 1.1.2, it is over 70% higher than in subclass 1.2.2, even though a below-median income level was used as the delimitation criterion in both cases. Another important feature of “surplus” countries is either a large amount of arable land per capita (subclass 1.1.1) or a highly intensive production system (subclass 1.1.2). Paradoxically, wealthy countries rely on quite extensive systems of agricultural production. Note however that many of them are located in both Americas, Australia and Central Asia, i.e. in regions with a relatively low population density. Also, it largely results from moderately favorable conditions for agricultural production which, in turn, is the consequence of these countries being located deep within large continents. Ultimately, the low population density (and the related low demand for food) and prosperity is what makes these countries net exporters of food, even at quite low levels of unit productivity (calculated in thousand kcal / ha UAA). Note that this subclass also includes European countries (Denmark, the Netherlands, Germany, France, Hungary, Poland, Lithuania) which meet the delimitation criteria (output greater than consumption and GDP per capita above median level) but are subject to different natural conditions. They have a high population density, an arable land per capita ratio fluctuating below 1 ha and a highly intensive production system.

A similar situation exists in subclass 1.1.2 countries where the arable land per capita ratio is low and production intensity is high. The subclass includes medium wealthy countries, mostly located in areas conducive to agricultural production. This could suggest the output surplus mainly results from natural conditions, high labor intensity of production processes, and moderate capital expenditure. However, even though output is greater than consumption, over 12% of the population of countries grouped in this subclass are undernourished. Once again, this shows that genuine solutions to food problems primarily depend on economic conditions.

Class 1.2 also includes other countries with diverse natural, geographic and economic conditions. However, the average per capita GDP is more than twice lower that the level recorded in class 1.1. Interestingly, despite a relatively high level of land productivity (expressed in thousand kcal / ha UAA), the agriculture is unable to fully address the food requirements. Per capita output is below the consumption level, and the net trade in agricultural commodity is negative. This could be explained by the small amount of arable land per capita which does not exceed 1 ha in any subclass. While the average level across the class is close to that recorded in subclass 1.1.2, the latter has a better land productivity. This means that in class 2.1 countries, the agricultural deficit is caused by relatively unfavorable geographic and natural conditions, demographic pressures, or both. Note however that despite these difficulties, the countries are committed to self-sufficiency in food. The average contribution of domestic production in the daily energy supply (in kcal per capita) is 86% across class 1.2 countries. For subclasses 1.2.1 and 1.2.2, the figures are 73% and as much as 92%, respectively. Once again, this shows the strategic importance of food and its role in ensuring internal security for particular countries. The global nature of today’s economy, including the agri-food sector, is particularly noticeable in class 1.2.1 countries which, though not self-sufficient in food, report the highest daily energy supply of all aggregates identified and a relatively small share of the undernourished population, which is due to a high level of prosperity. This is especially true for Middle East and North African countries where a desert or semi-desert environment dramatically hampers the achievement of food self-sufficiency but oil revenues make it possible to effectively address the shortfalls. Another situation was encountered in subclass 1.2.2 where a small average level of per capita GDP closes off exporting opportunities. Therefore, the daily energy supply is low and the share of the undernourished population exceeds 17%.

Class 2.1 includes only two countries: Bolivia and Rwanda. Their situation is unusual and quite controversial in ethical terms. In these countries, while the output expressed in kcal per capita exceeds the consumption level, the average daily energy supply is at one of the lowest levels and the share of the undernourished population exceeds 37%. Also, in accordance with the delimitation criterion, the daily energy supply does not fully address the local population’s demand. Contributing to this situation are the unfavorable natural and geographic conditions, largely resulting from the low levels of per capita GDP and the fact that both countries are located in landlocked areas deep within large continents. As shown by the analysis, even the large area of arable land per capita does not solve the problem as it fails to compensate for the low productivity of land. However, most worryingly of all, despite the agricultural shortcomings described above, these countries are net exporters of agricultural commodity in terms of both energy and economics. This can reasonably be expected to be an attempt to improve the economic performance by exporting agricultural raw materials. Nevertheless, the above also suggests these countries experience an extreme social imbalance.

A similarly disadvantageous situation is faced by class 2.2 countries, though they are characterized by an agricultural deficit. These include only poor countries, located mainly in Sub-Saharan Africa and Central Asia which are regions affected by natural farming handicaps. This results in the lowest intensity of agricultural production of all country groups identified. Also, despite the largest arable land per capita, this class reports the lowest daily energy supply which, in accordance with the class delimitation criteria, fails to address the energy demand. Moreover, these countries witness the largest share of the undernourished population which account for as much as over 40% of the total population.

## Discussion

This study revealed considerable territorial differences in agricultural production capacity. Similar conclusions were drawn by Porka et al. [42]. Based on a dynamic analysis, they discovered that the share of the world’s population living in countries with the highest production volume did not change significantly in 1962–2005, and was ca. 25% throughout that period. It follows from our analyses that most “global granaries” are wealthy countries which also experience favorable natural conditions. Considering the essence of agriculture -which boils down to the interaction between nature and human activity—the combination of both of these characteristics justifies the surplus capacity of these countries. Indeed, they enjoy agro-climate conditions, productive resources and high technology levels which favor the production of food. As emphasized by Chavas [52], these factors (in addition to price levels) are the key determinants of food production. At the same time, Bureau and Swinnen [53] note that wealthy northern countries, including the European Union, have considerably hampered the agricultural development of poor southern countries over the last decades, mainly by employing protectionist practices as a part of their trade policies. Export subsidies were the most important measures taken in that respect. But because of pressures from the WTO, recent reforms of the Common Agricultural Policy have mitigated the resulting distortion of the agri-food market. This is important for one more reason: as demonstrated by many researchers, including Tscharntke et al. [54], when considered globally, food insecurity is more a problem of food distribution than food production.

The conducted analysis proved that a slightly different approach to food consumption comparing with production needs to be adopted. Among the different factors on which consumption depends on are the wealth level of the society and income distribution [52, 55, 56]. Our study shows that the largest daily energy supply per capita is observed in countries with the highest GDP per capita and the smallest is observed in the poorest ones mainly from Sub-Saharan Africa and Central Asia. Note that ultimately, the dependence between the consumption level and national wealth is a bidirectional relationship. Johnes [57] demonstrated the existence of an individual relationship between food insecurity and mental health levels. Therefore, on a broader scale, the share of undernourished people means a generally low level of human and social capital which hinders economic development of a country. As indicated by Smith et al. [58], low levels of education, poorly developed social networks and low social capital all make food insecurity a much more likely experience. Moreover Luan et al. [59] proved that African countries’ suffer not only from a seriously unbalanced domestic economy which determines low food self-sufficiency, but also from wide disparities between the rich and the poor. That conclusion is not only true for poor countries; the same applies to disparities within the societies of highly developed countries. For instance, according to Borch and Kjærnes [60], 14 percent of the American lowest-income population suffer from food insecurity.

As shown by previous experience from countries around the world, famine was reduced as a consequence of both general economic growth and improved efficiency of agricultural production, including labor productivity. As noted by Headey, Alauddin and Rao [61], on a global scale, the improvement in labor productivity contributes about half to the increase in total productivity of agricultural inputs. It also played a key role in historical economic development by entailing a decrease in food prices [62]. The agricultural sector may provide a momentum for the entire economy; increasing the agricultural income by generating demand for non-agricultural products and services has a positive impact on the local economy and economic growth [63]. Also, Xinshen et. al [64] conclude that even though certain African countries have some potential for growth outside the agriculture, in many cases their industrial sectors are not likely to embark on the growth path (at least in the medium term). Moreover, non-agricultural growth is less effective in reducing poverty than agricultural growth because the later enables a greater contribution of the poor to the growth process. Considering the above, WTO and other organizations call for the liberalization of trade, including agri-food trade, as a lever of development for countries dealing with food problems [65]. However, as noted by Stiglitz and Charlton [66] and Whalley [67] trade liberalization is not an unconditional driver of economic growth and development; in the context of market imperfections, it does not necessarily have to result in an improvement of economic efficiency In developing countries, it is imperative to strengthen the internal competitiveness of the agricultural sector. In order for this to happen, adequate intervention measures must be taken. An important problem in the context of food security is to tell whether access to food (in adequate quantities and of an adequate quality) may be entirely left to free market forces. The experience of highly developed countries shows that as far as agri-food trade is concerned, free market mechanisms cannot be fully implemented. The problem is to determine the extent of market protection rather than to entirely abolish it. We therefore argue that a complete liberalization of agriculture is not a panacea for the global food system, and an in-depth further analysis must be carried out to address this problem.

## Conclusions

The purpose of this study was to determine the differences in both food security and food self-sufficiency across countries around the world. The typology was intended to support the objective defined in this paper which was to identify:

1. the countries which, due to favorable natural conditions and capital expenditure, are able to produce enough food to feed their own population and that of other countries,
2. countries who are unable to produce enough food but, due to their economic condition, are able to import food in quantities sufficient to ensure food security,
3. countries which, for various natural and economic reasons, face food problems.

As shown by this study, the degree to which food security is ensured with domestic supply varies strongly across the globe. This is the case in wealthy countries, usually located in scarcely populated areas with favorable conditions for agriculture (including North America, Australia, New Zealand, Kazakhstan) and in countries which, though characterized by a relatively small area of arable land per capita, demonstrate high production intensity (mainly European countries). These regions of the world report a high daily energy supply, do not experience any food problems and play a major role in global agri-food exports. Note however that the vast majority of countries seek the greatest possible food production autonomy because of the strategic importance of food. Examples include class 1.2 countries where the difference between production and consumption is 15% of the production volume. Nevertheless, international trade may largely offset the natural limitations. This is especially true for countries where even considerable capital expenditure is not enough to overcome the environmental barriers. Desert, mountain and arctic countries are affected by this phenomenon in its most extreme form. Note however that international trade instruments may be used only if a country holds enough financial resources to pay for imported goods, i.e. if it has a positive net trade in other products or services. This is the case for Middle East and North African countries as well for selected South American net importers of food products. In these regions, the share of the undernourished population is relatively low. The worst situation is experienced in groups 2.1 and 2.2, mainly composed of Sub-Saharan African and Central Asian countries which report a high share of undernourished population. Therefore, the ultimate basic conclusion from this study is that the food production volume (and food self-sufficiency, to a great extent) depends most on natural conditions, whereas economic conditions, primarily including national wealth, have the greatest impact on food consumption and security levels. Unfavorable natural conditions may be offset by foreign trade which, however, requires adequate amounts of capital to be allocated to agri-food imports. Therefore, especially as regards food insecure countries, it is essential to provide the agriculture sector with support focused on production efficiency improvements [68]. This is a way to ensure food self-sufficiency, at least to a partial extent. What also matters is the role of the international community in the general economic development of these countries, as necessary for a country’s ability to finance the import of food.

## Supporting information

S1 File Diagram of the research process. Source: own elaboration.
(PDF)

S1 Table Countries by classes and subclasses. Source: own elaboration.
(PDF)
