# Supplementary figures and images for: Food security and food self-sufficiency around the world: A typology of countries
Source: PLoS One. 2019 Mar 7;14(3):e0213448. doi: 10.1371/journal.pone.0213448 (PMC6407907; doi:10.1371/journal.pone.0213448)

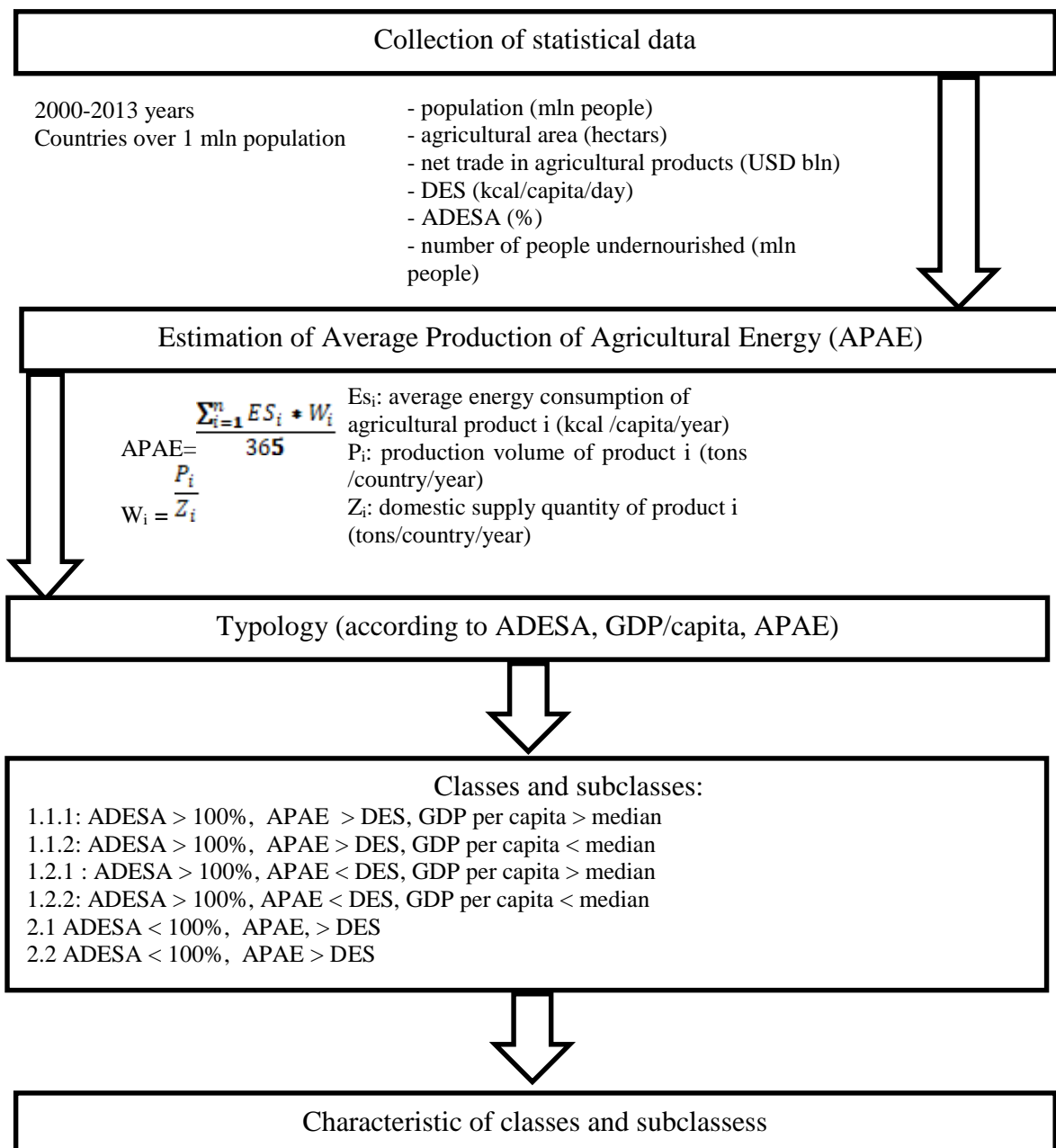

**S1 File. Diagram of the research process**

Source: own elaboration.

Supplement: S1 File — Source: own elaboration. (PDF) [file pone.0213448.s001.pdf]
