# Supplementary material for: Food security and food self-sufficiency around the world: A typology of countries
Source: PLoS One. 2019 Mar 7;14(3):e0213448. doi: 10.1371/journal.pone.0213448 (PMC6407907; doi:10.1371/journal.pone.0213448)
Supplement: S1 Table — Source: own elaboration. (PDF) [file pone.0213448.s002.pdf]

S1 Table. Countries by classes and subclasses

Class	Subclass	Country
1.1	1.1.1	Argentina
1.1	1.1.1	Australia
1.1	1.1.1	Canada
1.1	1.1.1	Czechia
1.1	1.1.1	Denmark
1.1	1.1.1	France
1.1	1.1.1	Germany
1.1	1.1.1	Hungary
1.1	1.1.1	Ireland
1.1	1.1.1	Kazakhstan
1.1	1.1.1	Lithuania
1.1	1.1.1	Malaysia
1.1	1.1.1	Netherlands
1.1	1.1.1	New Zealand
1.1	1.1.1	Poland
1.1	1.1.1	United States of America
1.1	1.1.2	Brazil
1.1	1.1.2	Bulgaria
1.1	1.1.2	Costa Rica
1.1	1.1.2	Ecuador
1.1	1.1.2	Guatemala
1.1	1.1.2	Indonesia
1.1	1.1.2	Lao People's Democratic Republic
1.1	1.1.2	Malawi
1.1	1.1.2	Mauritius
1.1	1.1.2	Myanmar
1.1	1.1.2	Paraguay
1.1	1.1.2	Republic of Moldova
1.1	1.1.2	Thailand
1.1	1.1.2	Ukraine
1.1	1.1.2	Uruguay
1.1	1.1.2	Viet Nam
1.2	1.2.1	Algeria
1.2	1.2.1	Austria
1.2	1.2.1	Azerbaijan
1.2	1.2.1	Belarus
1.2	1.2.1	Belgium
1.2	1.2.1	Chile
1.2	1.2.1	Colombia
1.2	1.2.1	Croatia
1.2	1.2.1	Dominican Republic
1.2	1.2.1	Egypt
1.2	1.2.1	Estonia
1.2	1.2.1	Finland
1.2	1.2.1	Greece

1.2	1.2.1	Iran
1.2	1.2.1	Israel
1.2	1.2.1	Italy
1.2	1.2.1	Japan
1.2	1.2.1	Jordan
1.2	1.2.1	Kuwait
1.2	1.2.1	Latvia
1.2	1.2.1	Lebanon
1.2	1.2.1	Luxembourg
1.2	1.2.1	Mexico
1.2	1.2.1	Norway
1.2	1.2.1	Panama
1.2	1.2.1	Peru
1.2	1.2.1	Portugal
1.2	1.2.1	Republic of Korea
1.2	1.2.1	Romania
1.2	1.2.1	Russian Federation
1.2	1.2.1	Saudi Arabia
1.2	1.2.1	Slovakia
1.2	1.2.1	Slovenia
1.2	1.2.1	South Africa
1.2	1.2.1	Spain
1.2	1.2.1	Sweden
1.2	1.2.1	Switzerland
1.2	1.2.1	The former Yugoslav Republic of Macedonia
1.2	1.2.1	Trinidad and Tobago
1.2	1.2.1	Tunisia
1.2	1.2.1	Turkey
1.2	1.2.1	United Arab Emirates
1.2	1.2.1	United Kingdom
1.2	1.2.1	Venezuela
1.2	1.2.2	Albania
1.2	1.2.2	Angola
1.2	1.2.2	Armenia
1.2	1.2.2	Bangladesh
1.2	1.2.2	Benin
1.2	1.2.2	Bosnia and Herzegovina
1.2	1.2.2	Burkina Faso
1.2	1.2.2	Cambodia
1.2	1.2.2	Cameroon
1.2	1.2.2	China
1.2	1.2.2	Côte d'Ivoire
1.2	1.2.2	Cuba
1.2	1.2.2	El Salvador
1.2	1.2.2	Gambia
1.2	1.2.2	Georgia

1.2	1.2.2	Ghana
1.2	1.2.2	Guinea
1.2	1.2.2	Guinea-Bissau
1.2	1.2.2	Honduras
1.2	1.2.2	India
1.2	1.2.2	Jamaica
1.2	1.2.2	Kyrgyzstan
1.2	1.2.2	Lesotho
1.2	1.2.2	Mali
1.2	1.2.2	Mauritania
1.2	1.2.2	Morocco
1.2	1.2.2	Mozambique
1.2	1.2.2	Nepal
1.2	1.2.2	Nicaragua
1.2	1.2.2	Niger
1.2	1.2.2	Nigeria
1.2	1.2.2	Pakistan
1.2	1.2.2	Philippines
1.2	1.2.2	Senegal
1.2	1.2.2	Sierra Leone
1.2	1.2.2	Sri Lanka
1.2	1.2.2	Sudan
1.2	1.2.2	Togo
1.2	1.2.2	Turkmenistan
1.2	1.2.2	Uganda
1.2	1.2.2	United Republic of Tanzania
1.2	1.2.2	Uzbekistan
1.2	1.2.2	Yemen
2.1		Bolivia
2.1		Rwanda
2.2		Botswana
2.2		Central African Republic
2.2		Chad
2.2		Congo
2.2		Democratic People's Republic of Korea
2.2		Haiti
2.2		Kenya
2.2		Liberia
2.2		Madagascar
2.2		Mongolia
2.2		Namibia
2.2		Tajikistan
2.2		Zambia
2.2		Zimbabwe

Source: own elaboration.
